# Elucidation of Essential Genes and Mutant Fitness during Adaptation toward Nitrogen Fixation Conditions in the Endophyte Azoarcus olearius BH72 Revealed by Tn-Seq

**DOI:** 10.1128/spectrum.02162-22

**Published:** 2022-11-23

**Authors:** Theresa Harten, Rolf Nimzyk, Vivian E. A. Gawlick, Barbara Reinhold-Hurek

**Affiliations:** a University of Bremen, Faculty of Biology and Chemistry, CBIB Center for Biomolecular Interactions, Department of Microbe-Plant Interactions, Bremen, Germany; b University of Bremen, Faculty of Biology and Chemistry, CBIB Center for Biomolecular Interactions, Nucleic Acid Analysis Facility (NAA), Bremen, Germany; Centre National de la Recherche Scientifique, Aix-Marseille Université

**Keywords:** endophytes, *Azoarcus olearius*, nitrogen fixation, Tn*5* transposon mutagenesis, essential genes

## Abstract

Azoarcus olearius BH72 is a diazotrophic model endophyte that contributes fixed nitrogen to its host plant, Kallar grass, and expresses nitrogenase genes endophytically. Despite extensive studies on biological nitrogen fixation (BNF) of diazotrophic endophytes, little is known about global genetic players involved in survival under respective physiological conditions. Here, we report a global genomic screen for putatively essential genes of *A. olearius* employing Tn*5* transposon mutagenesis with a modified transposon combined with high-throughput sequencing (Tn-Seq). A large Tn*5* master library of ~6 × 10^5^ insertion mutants of strain BH72 was obtained. Next-generation sequencing identified 183,437 unique insertion sites into the 4,376,040-bp genome, displaying one insertion every 24 bp on average. Applying stringent criteria, we describe 616 genes as putatively essential for growth on rich medium. COG (Clusters of Orthologous Groups) assignment of the 564 identified protein-coding genes revealed enrichment of genes related to core cellular functions and cell viability. To mimic gradual adaptations toward BNF conditions, the Tn*5* mutant library was grown aerobically in synthetic medium or microaerobically on either combined or atmospheric nitrogen. Enrichment and depletion analysis of Tn*5* mutants not only demonstrated the role of BNF- and metabolism-related proteins but also revealed that, strikingly, many genes relevant for plant-microbe interactions decrease bacterial competitiveness in pure culture, such type IV pilus- and bacterial envelope-associated genes.

**IMPORTANCE** A constantly growing world population and the daunting challenge of climate change demand new strategies in agricultural crop production. Intensive usage of chemical fertilizers, overloading the world’s fields with organic input, threaten terrestrial and marine ecosystems as well as human health. Long overlooked, the beneficial interaction of endophytic bacteria and grasses has attracted ever-growing interest in research in the last decade. Capable of biological nitrogen fixation, diazotrophic endophytes not only provide a valuable source of combined nitrogen but also are known for diverse plant growth-promoting effects, thereby contributing to plant productivity. Elucidation of an essential gene set for a prominent model endophyte such as *A. olearius* BH72 provides us with powerful insights into its basic lifestyle. Knowledge about genes detrimental or advantageous under defined physiological conditions may point out a way of manipulating key steps in the bacterium’s lifestyle and plant interaction toward a more sustainable agriculture.

## INTRODUCTION

Synthetic fertilizers have been employed for decades in industrial agriculture to secure productivity of arable crops at high levels; however, more environmentally friendly practices are demanded. Plant growth-promoting bacteria (PGPB) may be part of a concept for sustainable crop production. In contrast to symbiotic rhizobia inducing root nodules in legumes, endophytic bacteria colonize *Gramineae* like wheat, rice, and maize, highly relevant crops in global food production. Endophytic bacteria reside and multiply inside healthy plant tissues, establishing a tight interaction without threatening host viability (1). Their abilities to increase plant growth and health and to provide a nitrogen source to the host plant by the process of biological nitrogen fixation (BNF) are of increasing agronomic interest (2–4). These N_2_-fixing (diazotrophic) bacteria and their genetic makeup enabling beneficial host plant interaction are therefore of growing interest to current research.

Azoarcus olearius BH72 is a diazotroph belonging to the *Betaproteobacteria* subgroup of *Proteobacteria* (5) and has become one of the best-studied model endophytes, with several striking characteristics. (i) It supplies combined nitrogen derived from BNF to its host, Kallar grass (Leptochloa fusca, L. Kunth) (6). (ii) The ability of efficient root colonization extends under laboratory conditions to rice, an important model plant. (iii) Functional genomics, mainly genome comparisons (7), mutant analyses coupled with reporter gene expression, and transcriptomics, have provided new insights into pathways and mechanisms relevant for its endophytic lifestyle. Among them are type IV pilus-dependent adhesion and motility (8–10), endoglucanase-mediated entry (11), flagellum-dependent spreading (12), and type V (13) and type VI (10) protein secretion systems.

The endophytic lifestyle of strain BH72 appears to be interconnected to microaerobiosis—typical for waterlogged roots—and nitrogen fixation. In field-grown roots (6), pot experiments with field soil (14), and gnotobiotic cultures (15), nitrogenase genes are expressed in roots; moreover, several proven or suspected interaction-related genes are upregulated under microaerobiosis or nitrogen fixation conditions according to transcriptome studies (13, 16, 17). Therefore, it is of great interest to analyze the contribution of *A. olearius* genes under these culture conditions in more detail by a global approach. Thus, we established and carried out a Tn*5* transposon-based mutagenesis approach combined with high-throughput sequencing (Tn-Seq) for *A. olearius*.

Tn-Seq is a powerful technique to simultaneously characterize thousands of individual mutants and describe their abundance in a population by next-generation sequencing. By measuring the number of insertion sites tolerated in a certain gene, essentiality can be assigned (18, 19). Over the last few years, essential genomes have been described for well-studied model organisms like Escherichia coli (20, 21) and Pseudomonas aeruginosa (22, 23). With increasing accessibility and decreasing costs of high-throughput sequencing systems, additional reports related to a broader range of bacterial species have been presented. However, essential genome analyses of plant-associated and especially endophytic bacteria are scarce, like those of a few root endosymbionts (24–27) and the endophyte Herbaspirillum seropedicae (28). While epiphytic genetic traits of Azoarcus olearius DQS4^T^ by Tn-Seq have been described recently, no detailed results regarding gene essentiality or relevant pathways have been presented (29); it is not clear whether the reported results are due to environmental conditions in the microniche, which can be mimicked in pure culture, or whether they are specific for plant association. Thus, we present one of the first major essentiality reports for a well-characterized model endophyte. Since we aimed beyond essentiality to provide new insights into metabolic shifts in *A. olearius*, we additionally analyzed three characteristic growth conditions mimicking the environmental changes from free living to host colonization. Therefore, an *A. olearius* Tn*5* mutant master library was generated on complex medium and subsequently tested on defined synthetic medium while growing aerobically, microaerobically, or on N_2_. Mutant competitiveness highlighted the relevance of metabolic traits and putative metabolic burdens.

## RESULTS AND DISCUSSION

### Engineering the Tn*5PpilA* transposable element.

**FIG 1:**
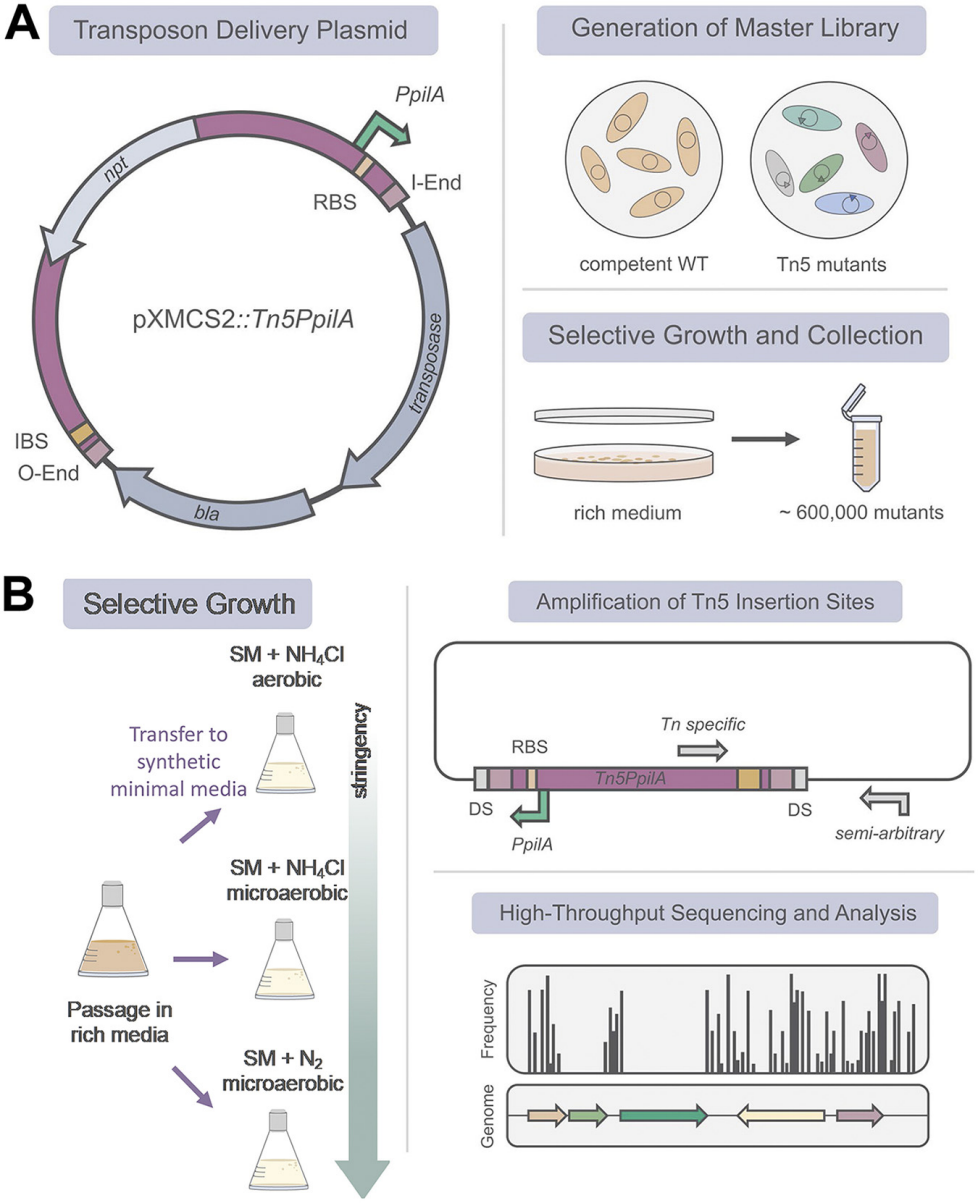
Strategy of transposon mutagenesis for library generation and Tn*5*-Seq. (A) Vector and protocol for master library generation and analysis. In five independent mutagenesis experiments, wild-type cells of *A. olearius* BH72 were transformed via electroporation and selected on nutrient-rich VM-malate-kanamycin plates. The number of CFU and therefore viable Tn*5* mutants was constantly high, with ~1.2 × 10^5^ CFU obtained for each sublibrary. For Tn-Seq-analysis, gDNA of this initial master library was extracted and Tn*5* insertion sites were amplified by a nested PCR introducing sample-specific indices and sequencing adapters. Amplicons covering individual Tn*5*-gDNA junctions were sequenced on an Illumina NextSeq platform for high-throughput analysis. Received reads were filtered according to the obligatory Tn*5* O-end sequence of every read derived from transposon insertion, trimmed, and aligned with the *A. olearius* genome. (B) Monitoring the effect of incremental shifting to N_2_ fixation conditions from complex medium. The Tn*5* mutant master library was first revived during a short passage on complex medium (VM-malate) and then subjected to one out of three different growth conditions on synthetic SM: aerobically or microaerobically on NH_4_Cl or microaerobically on N_2_. Tn*5* insertion sites of libraries prior and after selective growth were amplified and determined as outlined in panel A for the master library.

Creation of complex and therefore Tn-Seq suitable transposon libraries depends crucially on the chosen transposable element. Since the *A. olearius* genome displays a high GC content of 67.92% (7), a strategy was pursued based on Tn*5*, which does not show a bias toward TA insertion sites like the *mariner* transposon. Devoid of any insertion site requirements or strong regional preferences (30, 31), its derivates have been successfully applied in a variety of Tn-Seq studies (23, 32–34). Engineering of an *A. olearius*-specific transposon, *Tn5PpilA*, aimed at the following genetic properties. A medium-active, constitutive promoter, *PpilA* (8, 35), replaced at the transposon I end the *xylX* promoter of Caulobacter crescentus, which was not active in strain BH72 (not shown). This should allow initiation of transcription at the internal start site, to drive synthesis of RNA from the I end into its flanking genomic region (Fig. 1A and see Fig. S1 in the supplemental material). Depending on the target gene orientation, every Tn*5* insertion can be assigned an insertion orientation (i.e., sense or antisense). While antisense insertions disrupt transcription, sense insertions facilitate *PpilA*-driven transcription of downstream genes. The latter ensures functional integrity of operon structures; prevention of inhibitory polar effects, however, may lead to nonnative expression downstream of insertion. Analysis of randomly selected Tn*5* mutants by Southern hybridization of genomic DNA verified Tn*5PpilA* functionality: insertions occurred randomly and singularly in the *A. olearius* genome (Fig. S2).

### Overview of the Tn-Seq strategy and complexity of the transposon mutant master library.

Located on a suicide plasmid, the novel *Tn5PpilA* element facilitated the construction of an *A. olearius* Tn*5* mutant master library. A large number of Tn*5* mutants (~6 × 10^5^) was obtained for the final master library on complex, nutrient-rich medium, a key prerequisite for broad Tn-Seq experiments in *A. olearius* (Fig. 1A).

First, we pursued elucidation of putatively essential genes for growth on rich medium. The initial master library (five sublibraries) was subjected to Tn-Seq (Fig. 1A). Second, to disclose genes playing a role during change of lifestyle toward N_2_ fixation conditions, separate incremental shifts from aerobic complex medium to microaerobic growth on N_2_ were applied (Fig. 1B). Tn*5* mutant libraries were subjected to three different selective growth conditions on synthetic medium (SM): aerobically on NH_4_Cl and microaerobically on NH_4_Cl or N_2_ (Fig. 1B).

**FIG 2:**
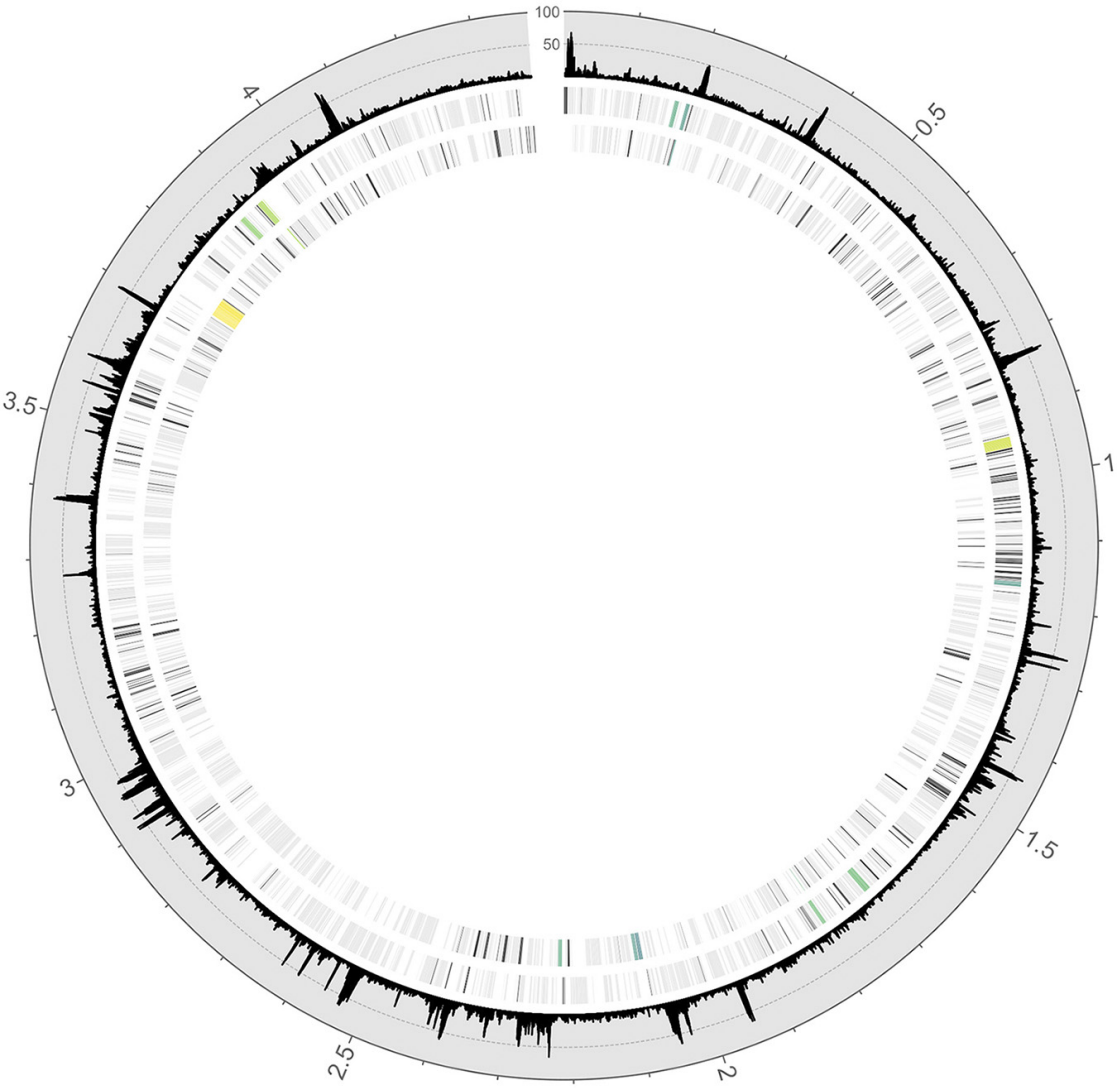
*A. olearius* genome-wide map of determined Tn*5* insertion sites and putatively essential protein-coding genes. From outer to inner track: (i) frequency of detected Tn*5* insertion sites with a bin size of 100 bp (those with the same insertion sites but with a different insertion orientation were only considered once), (ii) the genomic plus strand, and (iii) the genomic minus strand. Nonessential protein-coding genes are in light gray, putatively essential protein-coding genes are in dark gray, and consecutive essential protein-coding genes are in tones increasing from dark green to yellow for cluster sizes of *n* ≥ 6 essential genes.

For the Tn*5* mutant master library (Fig. 1B), Illumina sequencing yielded 138 million raw reads, of which 55 million (40%) aligned to the 4.4-Mbp *A. olearius* genome (see the read counts in Table 1). A high proportion of reads mapped to the transposon delivery plasmid. This observation has also been reported previously (34), employing the pXMCS2::Tn*5Pxyl* derivate. We identified the first genomic base of every read and converted it into its corresponding Tn*5* insertion site. By this means, the analytical focus was shifted from the Illumina sequencing-initiating outside end (O end) to the *PpilA*-holding inside end (I end). Out of 315,853 determined insertion sites, 183,437 were found to be unique (defined by position and orientation), displaying one insertion event every 24 bp on average. This represents a very high density, especially in comparison to a previous study performed with a related strain of *A olearius* (29). Their insertion events were not calculated, but they can be assumed to be much lower as only roughly 5 times fewer mutants were obtained. Visualization of insertion site frequencies (Fig. 2) in the genome did confirm transposon functionality and absence of severe insertion bias. Sizes of gaps between consecutive insertion sites were analyzed and 80% of gaps were shown to be ≤25 bp. However, three gaps of ≥8 kb were found, the largest one displaying a size of 9.7 kb.

**TABLE 1:** Sequencing data of analyzed Tn*5* mutant libraries

Library	No. of sequence reads	No. (%) of reads:		No. of unique insertion sites^*a*^	Insertion density (bp/insertion)
		Filtered and trimmed	Mapped to genome		
Tn*5* mutant master library (~600,000 insertion mutants)^*b*^	137,733,953	84,951,973 (62)	54,839,034 (40)	183,437	24
VM-malate library	80,319,605	48,025,799 (60)	36,508,175 (45)	105,421	42
SM + NH_4_Cl aerobic library	64,738,098	37,980,238 (59)	29,180,637 (45)	61,594	71
SM + NH_4_Cl microaerobic library	70,210,462	41,800,451 (60)	32,922,911 (47)	55,198	79
SM-NH_4_Cl microaerobic library	73,506,591	43,872,586 (60)	33,830,536 (46)	65,198	67

a The definition of unique insertion sites is given in Materials and Methods.

b Library size based on estimated no. of CFU grown on kanamycin-containing VM-malate agar plates.

### Identification strategy for putatively essential genes for growth on rich medium.

Representing 91.2% of its sequence, the *A. olearius* genome harbors 4,054 annotated genes (7). We found 157,054 (86%) of the insertion sites to be intragenic, displaying a set of 372 genes devoid of any insertion site, here classified as “non-hit” genes. In Tn-Seq studies, genes that do not tolerate any insertion sites are commonly classified as essential, providing that transposon libraries are saturated. Often this gene set is further extended by genes displaying a low density and/or a certain pattern of insertions. To avoid false positives, we pursued a relatively conservative strategy by applying the following stringent criteria given below in Materials and Methods. Briefly, Tn*5PpilA* insertions in the +1 frame of an open reading frame (ORF) were neglected (due to the promoter, Shine-Dalgarno [SD] sequence, and start codon at the end of the transposon), and insertions were not considered when located in the last 10% of an ORF or in the flanking region of an insertion-free gap covering ≥80% of ORF length. In total, this analysis revealed 616 genes as putatively essential, subdividing them into 564 protein-coding and 52 RNA-coding genes (Table S1).

**FIG 3:**
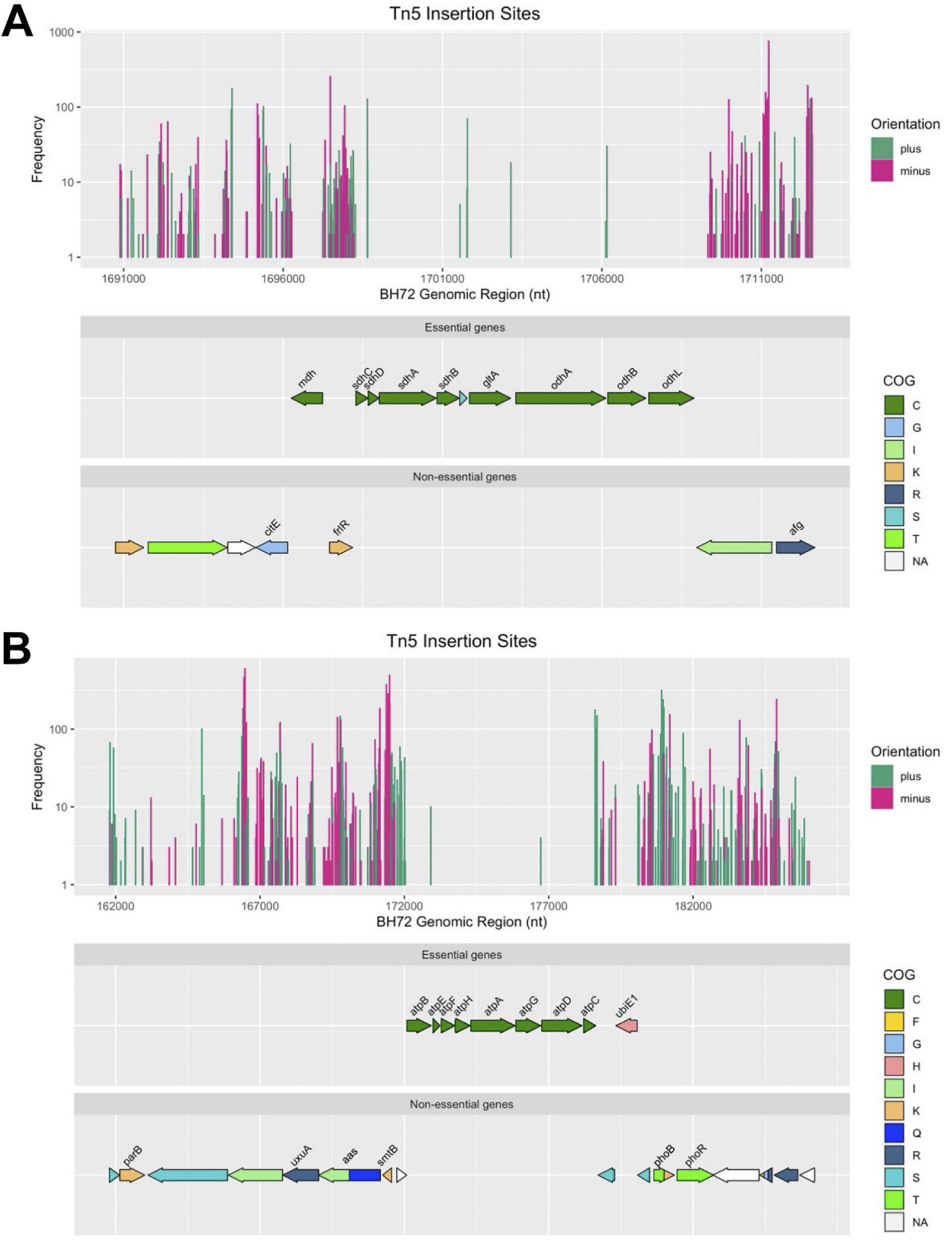
Genomic region encoding central enzymes of the TCA cycle (A) and the F-type ATPase (B) of *A. olearius*. From upper to lower panel: frequency of detected Tn*5* insertion sites of the respective genomic region and the determined insertion orientation. Genes labeled “plus” (green) and “minus” (pink) are defined as putatively essential and as nonessential, respectively, and their assigned COGs are shown.

Based on these findings, we reexamined Tn*5PpilA* performance in greater detail. With respect to insertion bias, comparisons between the essential gene set and the genome revealed only negligibly lower GC content of 66.72% on average, in comparison to 67.92% of the entire genome. Furthermore, the mean gene length of protein-coding genes of 928 bp was only slightly decreased compared to the genomic mean of 999 bp. Finally, we looked for transposon insertion orientation in relation to *PpilA* activity. Figure 3A exemplifies orientation restrictions upon gene essentiality for the genomic regions of *mdh*, *sdhCDAB*, *gltA*, and *odhAB*. Encoding central enzymes of the tricarboxylic acid (TCA) cycle, the respective genes were defined as putatively essential. In line with the transposon design, only sense insertions, particularly in intergenic regions of operons, were found to be viable, demonstrating the power of our Tn-Seq approach.

Our results defining 616 (15%) putatively essential genes are in accordance with previous reports, typically describing sizes of gene sets in the range 7 to 22% (21, 32, 36–39). In a Tn-Seq approach for a related *A olearius* strain (29), gene essentiality was not calculated. However, 443 genes represented non-hits (in contrast to 372 genes in strain BH72), but without filtering and in-depth analysis this cannot be compared to our data. Comparability is also constrained as they used two outwards-oriented promoters that are also different from our promoter, the kanamycin/neomycin resistance cartridge promoter (*kan/neo*), and the Salmonella *trp* promoter (P*trp*). Moreover, their lower Tn*5* insertion density makes it seem likely that essential genes would have been overestimated. Overestimation of essential gene set sizes is a common observation in transposon sequencing studies (40). Different statistical approaches for controlling the number of false positives are known (41–45). The prevailing method compares insertion densities. Normalized by length, insertion numbers or reads are expected to display a bimodal distribution. By applying a cutoff value, genes (41) or genomic windows (46) are tested for significance. Other authors employed a null model to test genes for the expected sum of insertion counts (42). The diversity of methods reflects the challenging aspect of transposon-based insertion analyses to fit a statistical model to a biological process that by its nature is not random. Independent of essentiality, DNA sites can be inaccessible for transposition due to their individual local structure, tightly associated protein complexes or reciprocal genetic effects (47).

We chose to elucidate gene essentiality based on the level of biological genome organization. Bacterial genes are known to be clustered in terms of functional association rather than being randomly scattered (48). The order of genes within a cluster can even resemble the sequence of reactions catalyzed by their products (49). Furthermore, genes involved in the same pathways are often found in operons, thereby coupling functionality and cotranscription (50). Thus, we asked for the probability of putatively essential genes in the *A. olearius* genome to form clusters by chance by applying a permutation-based Monte-Carlo approach. A set of 12 different clusters (Table S2) was found to be statistically significant, which comprised 138 out of the 616 putatively essential genes. The length of 10 clusters ranged from 6 to 11 genes, while the two longest ones were composed of 14 and 42 genes. Representatively, the eight ATP synthase-encoding genes are shown (Fig. 3B). Inside the cluster, only intergenic sense insertions were detected. Insertion bias became even more striking with regard to the respective promoter, displaying exclusively sense insertions. In line with the essential genome of Caulobacter crescentus (32), the gene upstream of *atpB*, *azo0152*, showed multiple sense insertions and is therefore defined as nonessential. In summary, out of the 564 putatively essential protein-coding genes, 138 were defined as essential for growth on rich medium because they formed statistically significant clusters in the genome.

### In-depth analysis of putatively essential protein-coding genes.

To provide an overview of the putatively essential gene set, we compared the representation of Cluster of Orthologous Groups (COG) categories among the 564 protein-coding genes to the overall genomic one. This extended gene number was included as the Monte Carlo approach was intended to find large gene clusters and thus indicate pathways, which might miss small gene clusters or single genes that might be important but not discovered by this approach. The results were in line with other essentiality reports (24, 26, 51), highlighting genes associated with core cellular function and viability as expected (Fig. S3). The highest enrichment was found in the category “translation, ribosomal structure and biogenesis” (category J). Forty-eight of the 52 genes were ribosomal component (*rps*, *rpl*, and *rpm*), translation initiation (*infC*, *infB*, and *infA1*), elongation (*tsf*, *tufAB*, and *fusA2*), and release factor (*prfA*) genes. All but one aminoacyl-tRNA synthetase gene were found to be essential. Intriguingly, *azo3203*, coding for methionyl-tRNA synthetase, was defined as nonessential. Manual inspection revealed multiple 3′-located insertion sites while its putative promoter and 5′ region remained undisrupted (Fig. S4A). We hypothesized that the C-terminal domain of Azo3203 was dispensable for protein function. Indeed, for the E. coli methionyl-tRNA synthetase (MetRS) a minimal enzymatic core was shown to be functional and stable (52, 53), while the appended domain contributes to dimerization and tRNA affinity (54). Azo3203 displays 54% identity to MetRS and possesses all annotated domains. Thus, provided regional high insertion densities, Tn-Seq enables dissection of polypeptide chains into essential and nonessential domains. We also identified *gatBAC* coding for an aspartyl-tRNA/glutamyl-tRNA amidotransferase as putatively essential. Since the *A. olearius* genome lacks an asparaginyl-tRNA synthetase-encoding gene, essentiality of the respective amidotransferase catalyzing the formation of asparaginyl-tRNA from aspartyl-tRNA has been expected and thus emphasizes data quality. Further essential genes were highly enriched within coenzyme transport and metabolism, amino acid transport and metabolism, nucleotide transport and metabolism, cell wall/envelope biogenesis, and intracellular trafficking (for details, see Fig. S3).

**FIG 4:**
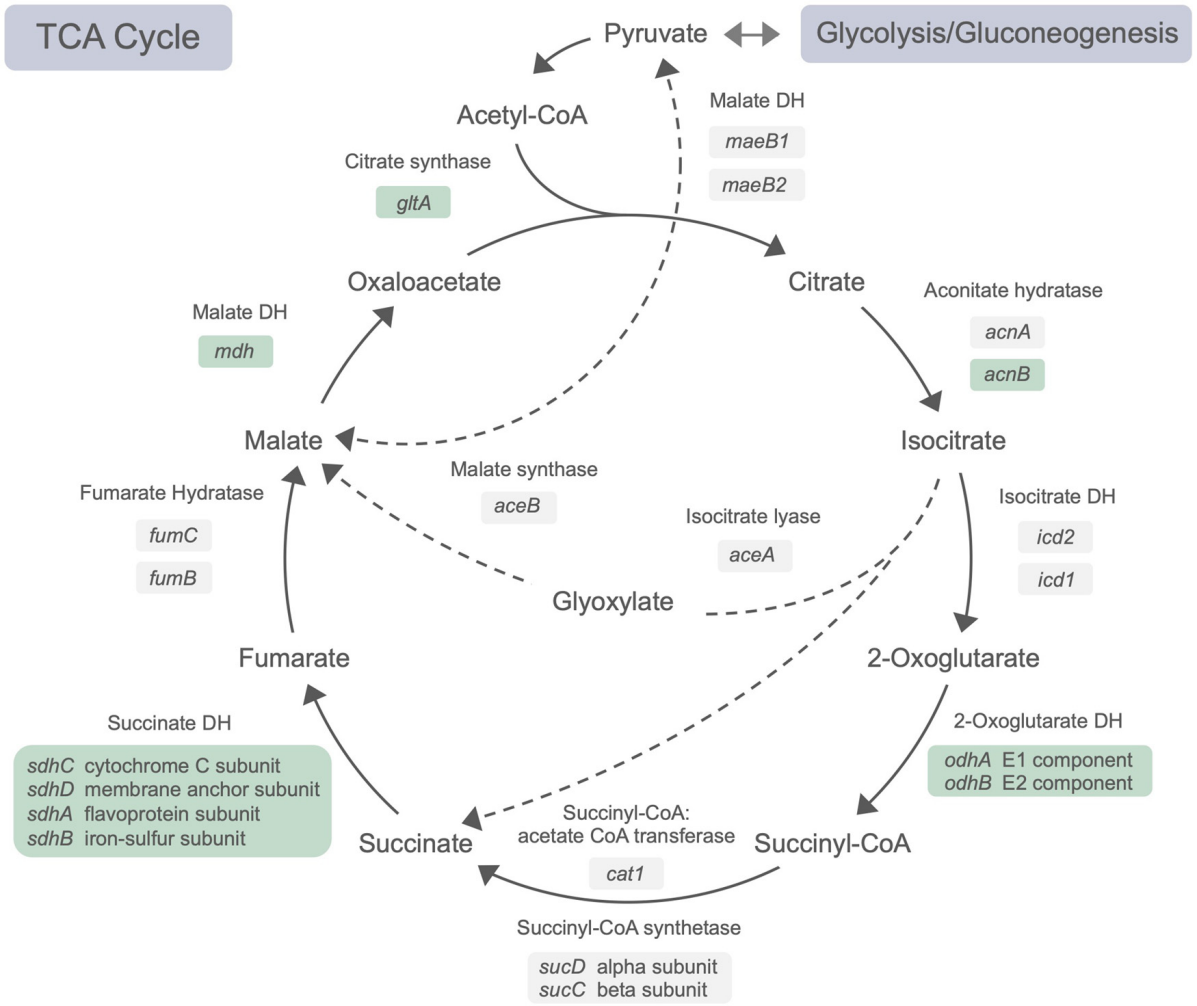
Schematic representation of the metabolic pathways of the TCA cycle. Genes encoding putative involved enzymes are given in italic. Genes described as putatively essential are further indicated in green. The dotted arrows represent pathways not directly assigned to the TCA cycle but interconnecting relevant intermediates.

As the permutation test found statistical significance for the gene clusters of *sdhCDAB*, *gltA*, and *odhABL*, we analyzed genes related to the TCA cycle in more detail (Fig. 4) and identified redundant and specific gene copies. Only one out of two aconitate hydratase-encoding genes, *acnB*, was defined as essential (Fig. S4B), concordant with reports for E. coli with AcnB as the major TCA cycle enzyme, while AcnA is related to iron and redox stress response (55). In contrast, isocitrate dehydrogenase genes *icd2* and *icd1* were found to be nonessential and physiologically redundant. Similar redundancy was apparent for the succinyl-coenzyme A (CoA) synthetase-encoding genes *sucDC* and the CoA transferase-encoding gene *cat1*, as well as genes encoding fumarate hydratases of classes I and II, *fumB* and *fumC*. Furthermore, *A. olearius* possesses the glyoxylate shunt-encoding genes *aceA* and *aceB*, bypassing two decarboxylation steps of the TCA cycle and thus providing metabolic flexibility for more balanced reductant levels under changing environmental conditions.

**FIG 5:**
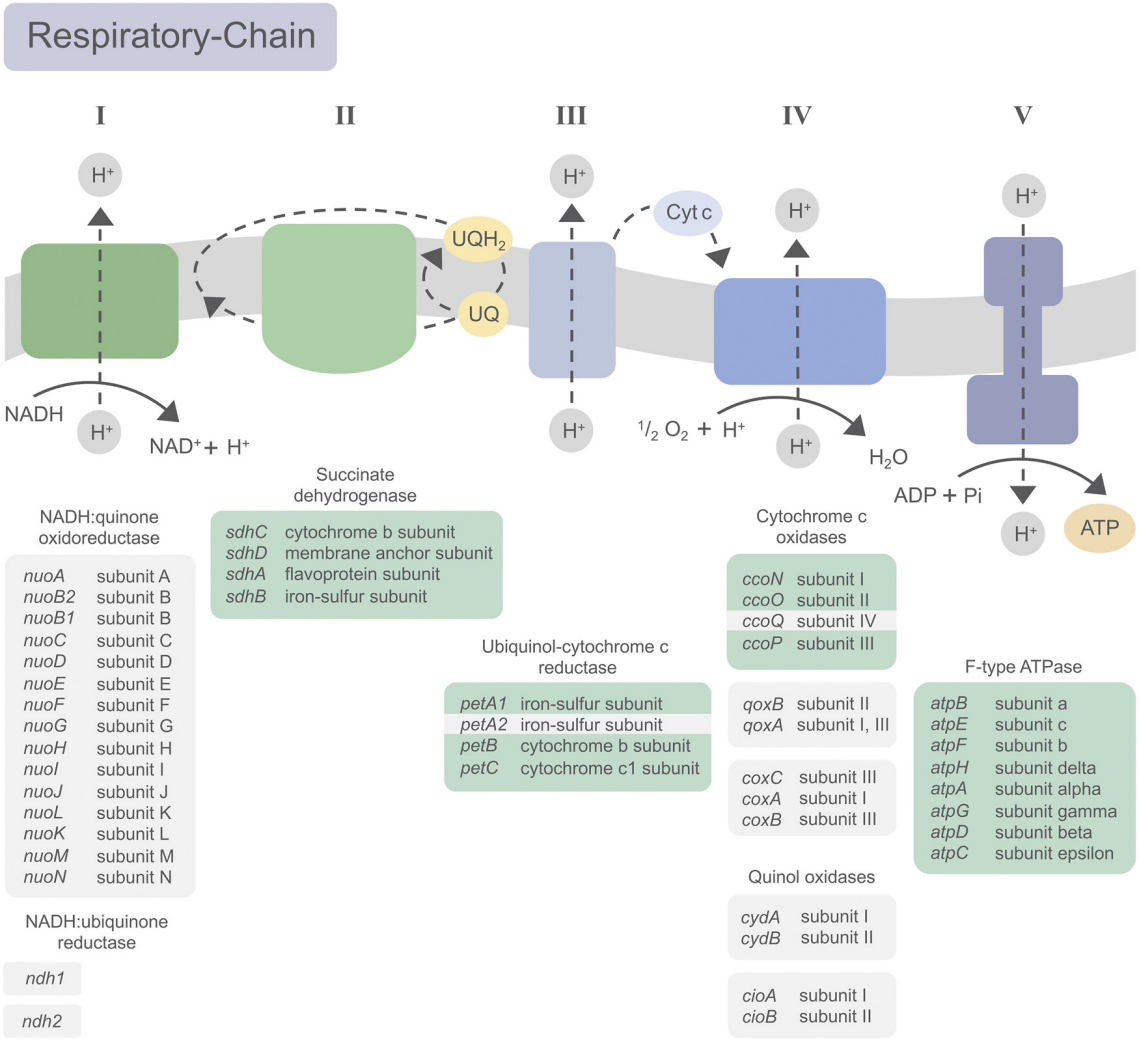
Schematic representation of the basic reactions of the respiratory chain and the catalyzing enzyme complexes. Genes encoding the putative involved enzymes are given in italic. Genes described as putatively essential are further indicated in green. The dotted arrows represent flows of cofactors and reductants.

Bacteria that naturally encounter fluctuating O_2_ levels are known for branched respiratory chains. This metabolic versatility can be based on different types of primary dehydrogenases and/or terminal oxidases (56). Essentiality of the F-type ATPase-encoding genes underlines the importance to generate energy by aerobic respiration in *A. olearius* (5). Among bacterial respiratory NADH dehydrogenases, *A. olearius* possesses not only the proton-pumping NDH-1 complex encoded by *nuo* genes but also two copies of nonpumping NDH-2-encoding genes (*ndh1* and *ndh2*), which appear to be redundant (nonessential) (Fig. 5). Thus, intracellular NADH/NAD^+^ ratios can be adjusted without affecting the strength of the proton motive force (PMF) and thereby respiratory energy conversion (56), allowing *A. olearius* to maintain metabolic flexibility upon changes in O_2_ and energy demands. With respect to the respiratory chain, five different gene clusters encoding either a cytochrome *c* oxidase (*cco*, *qox*, and *cox*) or a quinol oxidase (*cyd*, and *cio*) have been described for *A. olearius* (7, 13), suggesting redundancy. Transfer of e^−^ from the quinone/quinol pool to O_2_ can occur directly or via cytochrome *c* reduction by complex III. *A. olearius* is assumed to utilize quinol as well as cytochrome *c* oxidases (7, 57). Accordingly, the ubiquinol-cytochrome *c* oxidoreductase (complex III)-encoding genes *petA1BC* were found to be putatively essential. Unexpectedly, the *ccoNOQP* cluster encoding a high-affinity *cbb*_3_-type cytochrome *c* oxidase revealed discrepancies with our previous studies. Tolerating no or only sense insertions, the structural genes *ccoN*, *ccoO*, and *ccoP* were defined as putatively essential (Fig. S4C). However, a viable *ccoN* deletion mutant was constructed and analyzed in our laboratory, displaying no growth defect under aerobic conditions but with growth impeded by 2-fold under microaerobic conditions (13). Single inconsistencies in Tn-Seq studies have been reported and discussed for E. coli (20). Since in our case, local insertion densities were high, absence of genetic insertions by chance was highly unlikely. In addition, the small *ccoQ* gene (174 bp) did tolerate sense insertions and was therefore defined as nonessential, being perfectly in line with reports for Bradyrhizobium japonicum (58) and Rhodobacter sphaeroides (59). Disruption of the *ccoNOQP* cluster might lead to a mutant phenotype that is viable but not competitive under a limited O_2_ supply in a pool with other mutants.

### Overview of Tn*5* mutant libraries after selective growth.

In flooded rice roots, *A. olearius* naturally encounters microaerobiosis and low levels of combined nitrogen (13, 15). To mimic these shifts in a gradual manner, the Tn*5* master library was passaged in complex medium (VM-malate) and subsequently grown in (i) synthetic medium (SM) aerobically, (ii) microaerobically with NH_4_Cl, and (iii) microaerobically on N_2_. Tn*5* library composition before and after selective growth was analyzed as stated above. Illumina sequencing yielded 65 to 80 million raw reads per condition. Of these, 29 to 37 million (45 to 47%) aligned to the reference genome (Table 1). Library passage after freezing led to a 43% reduction of the mutant pool, with 105,421 unique insertion sites. Selective growth conditions led to further reduction by 38 to 48%: we determined 61,594 (i), 55,198 (ii), and 65,198 (iii) unique insertion sites, respectively. Insertion densities remained moderately high, displaying one insertion every 42 bp on average after library passage and every (i) 71 bp, (ii) 79 bp, and (iii) 67 bp after selective growth.

**FIG 6:**
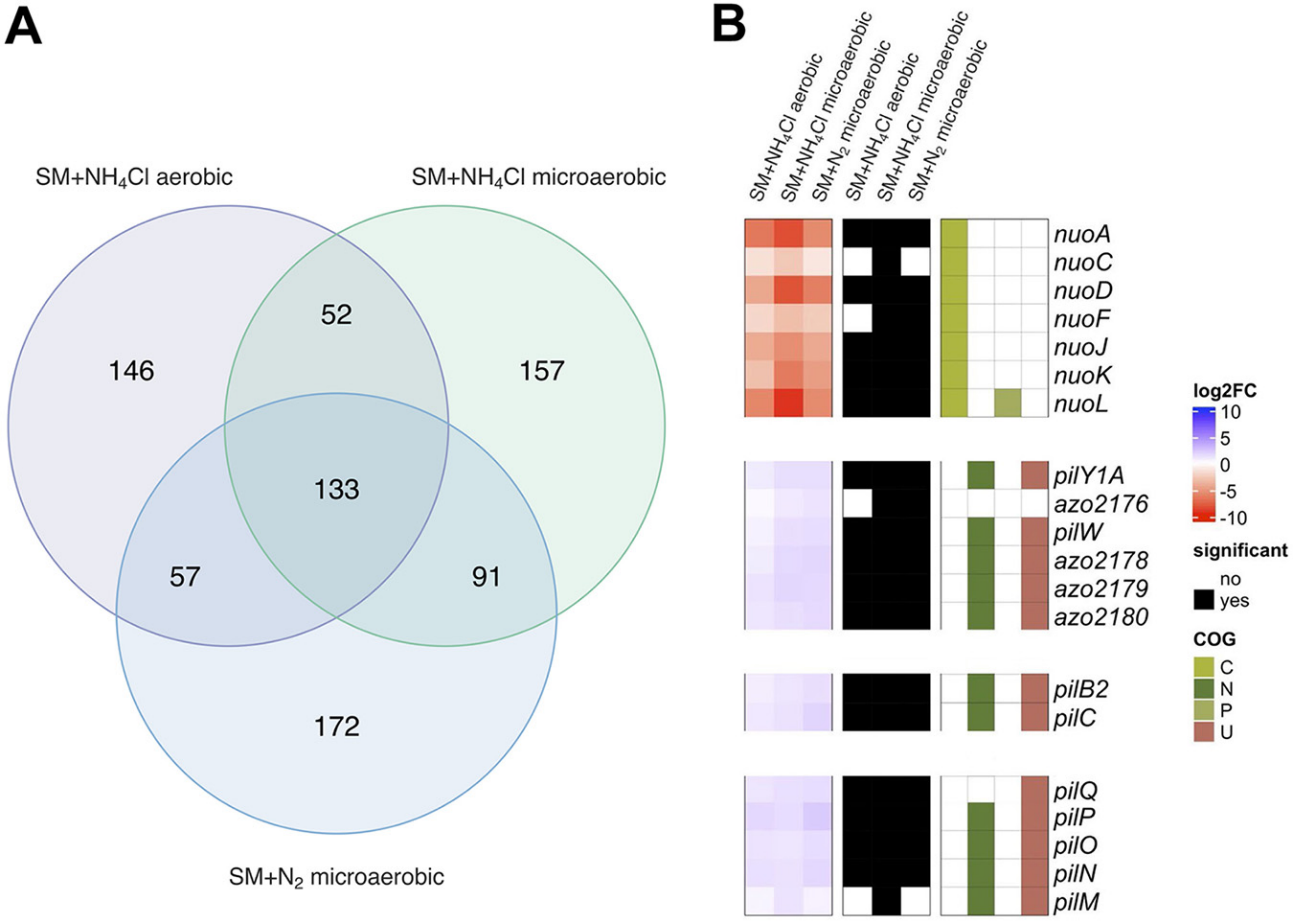
Comparison of genes identified as significantly depleted or enriched upon mutation during selective growth. (A) Quantitative overview based on the numbers of identified genes unique to or shared by the tested conditions. (B) Heat map of selected loci of genes identified as significantly depleted or enriched upon mutation. The level of change in abundance compared to the master library is shown as the negative (depleted) and positive (enriched) log_2_ fold change (FC), and genes found to be significant (black) or nonsignificant (white) and their assigned COGs are shown.

Subsequent analyses were carried out using the DESeq2 package (60), which identified 388 (i), 433 (ii), 453 (iii) genes to be significantly enriched or depleted upon mutation. A set of 133 genes was shared for all three conditions, while the number of genes uniquely enriched or depleted increased with prevailing stringency (Fig. 6). Visualization of mutant enrichment and depletion particularly revealed clusters of genes with similar fitness effects upon mutation (Fig. S5).

### Pilin and cell envelope mutants are enriched in all three selective growth conditions.

Among the gene clusters showing similar fitness effects were three clusters encoding type IV pilus biogenesis (*pilY1A*, *pilW*, *azo2178*, and *pilB2C*) and type IV pilus assembly (*pilNOPQ*) proteins. Mutations in all of these genes led to an enrichment of the respective mutants under all tested conditions, displaying a trend of increasing fold changes toward nitrogen fixation. Type IV pili are important for endophytic colonization, enabling effective attachment to and motility by twitching inside the host (8–10). Recent RNA-Seq studies further emphasized the need of diazotrophs to be mobile by demonstrating upregulation of the type IV pilus machinery during nitrogen fixation (17). Tn-Seq and RNA-Seq revealed a striking overlap of genes found to be significant (*pilY1A*, *pilW*, *azo2178*, *pilOPQ*, and *pilAB*). Type IV pili go through ATP-consuming cycles of rapid extension and retraction for twitching (61), which is likely to entail a metabolic burden and thus a competitive advantage in liquid medium when the genes are lost.

Several additional large clusters of genes appeared to be not only dispensable but even detrimental for competition in all three liquid cultures, as mutants were enriched (Fig. S5). They encoded mainly proteins putatively involved in cell envelop biogenesis and modification. Cluster *azo2674* to -*2697* as well as clusters *azo3189* to *-3193* and *azo3253* to *-3289* contain many genes related to lipopolysaccharide and O-antigen synthesis, transport, and modification or other cell envelope-related modifications of polysaccharides. Enrichment of the mutants suggests that also cell envelope-related modifications may bear a competitive disadvantage in synthetic medium due to increased metabolic burden. However, cell envelop composition is important to cope with environmental stress and during infection of hosts (62), and particularly lipopolysaccharides play major roles during host-pathogen interactions (63). In the endophyte Herbaspirillum seropedicae, lipopolysaccharide mutants show strongly decreased maize root colonization (64). It is remarkable that probably accessory genomic regions important for the plant-associated lifestyle appeared to decrease fitness/competitiveness in pure culture.

### Respiratory NADH dehydrogenase mutants are depleted under selective growth conditions.

Another noticeable cluster was composed of 7 out of the 14 NDH-1-encoding *nuo* genes. Upon mutation, five (*nuoA*, *nuoD*, and *nuoJKL*) of these were significantly depleted under all three tested conditions. As stated before, *A. olearius* is assumed to employ NDH-1 as well as NDH-2 for aerobic growth on rich medium. However, Tn-Seq indicates NDH-1 to be the main respiratory NADH dehydrogenase when growing in synthetic medium. Interestingly, mutant depletion was most pronounced under microaerobiosis (Table S3). In concordance with a global expression analysis detecting *nuoJKL* to be exclusively upregulated under microaerobiosis (13), our data indicate a major role of NDH-1 under O_2_-limiting conditions. Employing two functionally and metabolically different respiratory NADH dehydrogenases might be rewarding in several ways, such as a problematic NDH-1 activity to considerably contribute to reactive oxygen species (ROS) production (65–67), balancing intracellular NADH/NAD^+^ ratios by reoxidizing excess NADH by NDH-2 (68, 69). This unique property seems to be highly important during microaerobic growth since only then did Tn-Seq reveal *ndh1* mutants to be significantly depleted (Fig. S4).

### Mutants with mutations in protection and repair mechanisms against ROS are depleted during aerobiosis.

Analysis of mutants exclusively altered under aerobiosis on synthetic minimal medium revealed genes whose functions are related to protection and repair mechanisms against ROS. Global expression analysis upon the shift to microaerobiosis analogously described downregulation of respective genes (13). Among these were genes that Tn-Seq described as putatively essential for aerobic growth on rich medium, like *ccmC* coding for a heme chaperone, *lipA* coding for antioxidants, and *rodA* related to peptidoglucan formation. Management of ROS levels can be achieved by specialized enzymes/chaperones or in case of RodA indirectly by cell wall adjustments.

Three enzyme classes directly related to ROS reduction are superoxide dismutases, catalases, and peroxidases (70). Such mutants were depleted exclusively under aerobic conditions and not under microaerobic conditions on minimal medium, emphasizing oxygen stress for aerobic cells. Of two annotated *A. olearius* isoenzymes encoding a superoxide dismutase, *azo1466* was found to be putatively essential even on complex medium, underlining the importance of this enzyme type in comparison to the others described below. Mutant *ahpC*, *bcpI* and *ccp* genes, all coding for different peroxidases, were significantly depleted after aerobic growth. For E. coli, high-affinity Ahp was shown to play a major role in reducing low concentration of oxygen peroxide (H_2_O_2_) (71). E. coli *bcp* mutants were more sensitive to both H_2_O_2_ and organic peroxides (72). Ccp peroxidases are present in many Gram-negative bacteria (73) and are important for defense against peroxide stress (74).

Interestingly, mutations in the ferric uptake regulator *fur* were also found to be exclusively and severely depleted after aerobic growth. Transcriptional regulation by Fur is known to be directly or indirectly linked to challenges in defense against ROS (75). Disturbed iron homeostasis due to *fur* disruption is expected to increase ROS levels produced by the Fenton reaction, thereby leading to serious cell damage (76). Mutational studies in E. coli showed high levels of DNA damage (77), while in P. aeruginosa altered expression rendered cells more sensitive to ROS (78). Tn-Seq analysis confirmed the need of *A. olearius* to manage iron and ROS levels upon aerobiosis and outlines an important role of a set of distinct peroxidases.

### Tn-Seq reveals metabolic adaptations to microaerobiosis.

Previous transcriptome studies on microaerobiosis pointed out increased activity of TCA cycle-related anaplerosis and acetyl-CoA metabolism (13). Replenishment of intermediates was interpreted as the need for maintenance of metabolic flux and energy generation upon O_2_ limitation. Concordantly, Tn-Seq analysis revealed significant mutant depletion of *paaJ2*. PaaJ2-catalyzed formation of acetyl-CoA may relate to energy-generating butanoate metabolism (13). However, PaaJ2 activity is also involved in valine, leucine, and isoleucine degradation. Accordingly, *livM4* and *livK2* mutants encoding a branched-chain amino acid transport system were depleted. Being converted into acetyl-CoA/succinyl-CoA, degradation of these amino acids probably contributes to energy generation. Glutamate and aspartate can be directly converted into the TCA cycle intermediates 2-oxoglutarate and oxaloacetate, respectively. The importance of amino acid metabolism was further supported by additional mutants. Degradation of certain amino acids, such as glutamate and aspartate, probably contributes to energy generation. Mutants with mutations in the transport system-encoding genes *glnM* and *glnH* were also found to be depleted. Degradation of amino acids as an energy source is further supported by severe depletion of *pepD* mutants deprived of an encoded dipeptidase. While lack of NagL1 led to considerable mutant enrichment under aerobic conditions and is therefore expected to be dispensable only under this condition, *nagI* mutants were depleted under nitrogen fixation. Both enzymes are associated with amino acid metabolism and catalyze successive formation of the TCA cycle intermediate fumarate. The anaplerotic pathway seems to become more relevant with O_2_ limitation and lack of combined nitrogen. Also highly depleted under nitrogen fixation are *gabD2* mutants. The encoded succinate-semialdehyde dehydrogenase contributes to anaplerosis by formation of succinate.

Expression analysis of the response to microaerobiosis not only revealed enhanced activity of anaplerotic flux but also revealed enhanced activity of the TCA cycle-modifying glyoxylate shunt (13). The latter is facilitated by the activity of isocitrate lyase and malate synthase encoded by *aceA* and *aceB*, respectively. Based on upregulation of *aceA*, formation of glyoxylate and succinate was assumed to be enhanced. Interestingly, RNA-Seq indicated high activity of AceB under nitrogen fixation, producing malate from glyoxylate (17). This finding could be confirmed by Tn-Seq describing depletion of *aceB* mutants under nitrogen fixation. We hypothesize a shift of the metabolic flux from the decarboxylating arm of the TCA cycle to the glyoxylate shunt, with microaerobiosis climaxing upon nitrogen fixation. The latter would explain why depletion of *aceB* mutants could not be observed under microaerobic conditions. Balancing flux between both metabolic pathways is not an all-or-none switch (79). While the glyoxylate shunt becomes more relevant under microaerobiosis, gene inactivation can be still compensated for by the main sequence of the TCA cycle. However, with increasing flux during nitrogen fixation, *aceB* mutation is likely to elevate glyoxylate concentrations to the level of toxicity (80). The assumed gain of metabolic versatility is on the one hand based on net formation of malate enabling enhanced carbohydrate biosynthesis by gluconeogenesis (79) and on the other hand based on decreasing NADH levels generated by the TCA cycle (81). The latter might be an additional strategy to the described employment of alternative NADH dehydrogenases for balancing the NADH/NAD^+^ ratio upon the shift to microaerobiosis and nitrogen fixation.

The relevance of the glyoxylate pathway in host infection has been described for several species, including the plant pathogens Rhodococcus fascians and Xanthomonas campestris (82). For the human pathogen Pseudomonas aeruginosa, its role was even expanded to respiratory regulation and ROS generation (83). Direct proof for a significant role during rice root infection for *A. olearius* is still missing. However, cell-density-dependent modulation of *aceA* on conditioned supernatants was found compared to standard growth conditions (35), which are likely to prevail at roots.

### Nitrogen fixation and regulation mutants depleted during BNF.

Differential expression upon diazotrophic growth has been extensively studied in *A. olearius*, mainly by microarray and RNA-Seq approaches (16, 17). In support of our data quality, Tn-Seq revealed a high proportion of genes as significantly depleted that are known to be upregulated and important during BNF. One obvious gene cluster, harboring a multitude of these genes, is the *nif* cluster (*azo0512* to *azo0562*). Convincingly, mutants of the two master regulator-encoding genes *rpoN1 nifA* and the nitrogenase-encoding genes *nifHDK* were significantly depleted. Furthermore, mutants with mutations in genes associated with nitrogenase maturation and stabilization (*nifM* and *nifP*), FeMoco (FeMo cofactor) biosynthesis (*nifY1*, *nifZ*, *nifW*, *nifU*, *nifN*, and *nifE*), and electron transport (*nifF1* and *fprA*) were depleted. In line with previous expression analyses, Tn-Seq revealed severe mutant depletion for *rnfA1* and *rnfD1* encoding components of the Rnf1 complex for ion transport-coupled transfer of electrons to nitrogenase. Expression of *rnfA1* and *rnfD1* is positively regulated by NifA during nitrogen fixation, *rnfA1* being highly expressed during BNF (16, 84). Consistent with Tn-Seq, an *rnfA1* deletion mutant (84) showed significantly reduced generation time compared to the wild-type strain.

Mutants with mutations of *glnB* and *glnD* encoding a P_II_-like signal transmitter protein and a putative P_II_ protein uridylyltransferase, respectively, were also depleted. *A. olearius* possesses three functional P_II_-like signal transmitter proteins, of which GlnK and GlnB are differentially modified (uridylylated) in response to combined nitrogen limitation. Uridylylation of P_II_ proteins is often catalyzed by GlnD (85, 86). Neither GlnK nor GlnB is essential for BNF in strain BH72; however, the double mutant showed impaired growth on N_2_, ammonium, or nitrate as the sole nitrogen source (87). Enzyme assays revealing significantly reduced glutamine-2-oxoglutarate aminotransferase (GOGAT) suggested dysfunctional ammonium assimilation. Regulatory functions of the ammonium-assimilating enzymes by P_II_ proteins are well known: GlnB modulates glutamine synthetase (GS [the *glnA* product]) activity via an adenylyltransferase (ATase [the *glnE* product]) in several *Proteobacteria* (88, 89), the gene *glnA* being upregulated in *Azoarcus* during BNF (16). Additionally, GlnB modification influences the phosphorylation status of the NtrBC two-component system (TCS), thereby indirectly regulating expression of operons related to nitrogen fixation and/or assimilation (89). Tn-Seq-detected depletion of *glnB* mutants provides additional evidence for a regulatory role of this P_II_ protein during competitive diazotrophic growth in *A. olearius*. Whether the mode of action relates to ammonium assimilation or NtrBC-based regulation remains open.

### Tn-Seq suggests the importance of the histidine kinase NtrY and cell wall homeostasis during BNF.

Mutation of *ntrY* encoding a histidine kinase also led to mutant enrichment upon diazotrophic growth. The NtrYX TCS is widespread among *Alpha*- and *Betaproteobacteria* (90). While Tn-Seq described *ntrX* as putatively essential, *ntrY* mutation exclusively led to significant mutant enrichment upon nitrogen fixation. Essentiality of NtrX has been reported for Sinorhizobium meliloti and Brucella species (91). However, why the lack of a functional NtrY sensor in *A. olearius* led to increased mutant abundance under free-living diazotrophic growth remains an open question. Since *ntrX* is known to display pleiotropic effects (91) and is putatively essential for aerobic growth in *A. olearius*, the NtrYX TCS might be involved in other processes than nitrogen fixation, or NtrY might be functionally redundant due to possible NtrYX/NtrBC cross talk (84). It is involved in regulation of symbiotic root nodulation and nitrogen fixation (92–94) and was recently associated with cell envelope function (95).

Congruently, mutations in several genes associated with cell wall homeostasis led to distinct depletions after aerobic/microaerobic growth. Strikingly, no such effect could be observed upon BNF. Among these genes was *daaA*, encoding a d-alanine transferase. In comparison to l-amino acid aminotransferases, this class of enzymes transaminates only d-amino acids (96). They are required by bacteria for the synthesis of d-glutamic acid and d-alanine, which are important constituents of the cell wall and precursors for other d-amino acids (97). Also, mutation of *dacC* and *pbpG*, both encoding a d-Ala–d-Ala carboxypeptidase (DD-CPase), led to severe depletion upon aerobiosis/microaerobiosis. Enzymes of this class remove C-terminal d-alanyl residues from cell wall precursors, thereby positively controlling the extent of peptidoglucan cross-linking (98). Another depleted mutant functionally linked to cell wall homeostasis had a mutation in *cvpA* coding for an inner membrane protein (99). Interestingly, these findings are corroborated by RNA-Seq, demonstrating significant suppression of *daaA* and *dacC* in *A. olearius* during BNF (17). Bacterial modification of peptidoglycan plasticity is a well-known strategy to cope with environmental challenges (100). Endophytic nitrogen fixation might have other demands on cell wall composition like its free-living counterpart. Detected redundancy (Tn-Seq) and genetic suppression (RNA-Seq) of cell-wall-modulating enzymes might indicate the need for a more porous and less rigid envelope enabling diffusion/transport of gaseous and low-molecular-weight molecules. For E. coli, DD-CPase-encoding genes were shown to be nonessential for cell viability. However, deletion mutants revealed increased permeability and physiological alterations of the cell envelope (101). In accordance, integrity of *Bradyrhizobium* species DD-CPase-encoding genes was shown to be directly related to peptidoglucan cross-linking and even host colonization (102).

### Validation of candidate genes by mutants generated by site-directed mutagenesis.

To support the findings of our global analysis (Table S3), mutants that were created by site-directed mutagenesis were tested. Among highly depleted genes during nitrogen fixation, structural genes of nitrogenase had already been analyzed previously and were found to be essential for growth on N_2_ (103). Also, Tn*5* mutants with transcriptional activator NifA mutation were significantly reduced; mutation in *nifA* proved that it indeed encodes the essential transcriptional regulator of *nif* genes as the mutant was only able to grown in the presence of combined nitrogen not on N_2_ (104). Another mutant specifically depleted under BNF conditions had mutation in the *rpoN1* gene, encoding an alternative sigma factor. An in-frame deletion mutant was created and tested for growth on N_2_ in the oxygen-controlled bioreactor at 0.3% dissolved O_2_. While in triplicate experiments, the wild-type strain BH72 showed a mean generation time of 2.17 ± 0.06 h, but mutant BH Δ*rpoN1* did not grow at all, and even with addition of glutamate it reached no more than an optical density at 578 nm (OD_578_) of 0.06 within 22 h after inoculation with an OD_578_ of 0.003, indicating the sigma factor is highly important for nitrogen fixation (Fig. S6).

We further selected a gene depleted during aerobic growth in synthetic, ammonium-containing medium. To validate a competitive disadvantage of the transcriptional regulator Fur, a mutant with a gene inactivation mutation in the respective gene *azo2578* was created by plasmid integration mutagenesis. Competition experiments under the above conditions in liquid medium over roughly 20 generations revealed that the mutant was indeed significantly depleted compared to the wild type (37.9% ± 5.36%; *P* = 0.0052) (Fig. S6).

In summary, here we have demonstrated successful application of Tn*5-*Seq to obtain mutant libraries with very high insertion density, allowing us to examine not only gene essentiality but also operon and promoter structure and essential protein domains of an endophyte. In several cases, we were also able to discern whether a gene copy among similar genes might be most important for the function, as in case of the aconitate hydratase gene *acnB.* Although Tn-Seq has been applied to a related strain, *A. olearius* DQS4^T^ (29), these features had not been explored. Our approach also had the advantage that no excessive growth was required before the specific conditions were tested or again before DNA extraction, as previously done (29) leading to least additional bias in mutant quantification. We defined for the first time in such a global approach which genes and pathways contribute to fitness under different oxygen concentrations in synthetic medium and during nitrogen fixation and validated functions for several mutants generated by site-directed mutagenesis. It is striking that genes involved in the type IV pilus machinery and bacterial envelope modification were found to confer a competitive disadvantage in pure culture. As they are required or suspected to be involved in successful endophytic establishment, it is likely they have been retained in the endophytic accessory genome to maintain the endophytic lifestyle.

## MATERIALS AND METHODS

### Bacterial strains and standard growth conditions.

Escherichia coli DH5α strains were grown in Luria-Bertani medium (105) at 37°C. Kanamycin and ampicillin were added to medium or agar plates at final concentrations of 30 μg/mL and 150 μg/mL, respectively. Azoarcus olearius strain BH72 (106) and its mutant strains were cultivated aerobically at 37°C in modified VM-malate medium (5), deploying 0.3% peptone instead of beef extract and biotin. VM-malate medium contained the following (per liter): dl-malic acid, 5.0 g; KOH, 4.5 g; KH_2_PO_4_, 0.6 g; K_2_HPO_4_, 0.4 g; MgSO_4_·7H_2_O, 0.2 g; NaCl, 1 g; CaCl_2_, 0.02 g; MnSO_4_·H_2_O, 0.01 g; Na_2_MoO_4_·2H_2_O, 0.002 g; Fe(III)-EDTA, 66 mg; NH_4_Cl, 0.5 g; yeast extract, 1 g. Selective growth was carried out in SM (107) without biotin supplemented with 0.5 g/L NH_4_Cl or on N_2_ in an oxygen-controlled 2-L bioreactor (Infors AG, Bottmingen, Switzerland). For microaerobiosis, oxygen concentrations were set to 0.6%. The antibiotics applied were kanamycin and ampicillin at 30 μg/mL. Kanamycin-resistant mutant BH2578 was generated by plasmid integration mutagenesis using plasmid pK18GGST, as described previously (108), with an insertion site at bp 139 of *azo2578*. Gene *rpoN1* was inactivated by in-frame deletion (bp 28 to 1389) to generate mutant BH Δ*rpoN1* (109). For competition experiments of the wild type and mutants from site-directed mutagenesis, two strains were mixed in equal proportions and grown in liquid medium for roughly 20 generations. Ratios of the mutant and wild type were determined by plating and differentiating them by antibiotic resistance, as previously described (108), before and after the competition experiment; three independent experiments were carried out.

### Construction of transposon delivery plasmids.

The transposon delivery plasmid pXMCS2::Tn*5Pxyl* (32) was genetically modified at the transposon I end and O end. The I-end-located *xylX* promoter of Caulobacter crescentus was replaced by the *A. olearius pilA* promoter (8) via standard molecular DNA techniques (105). Briefly, two desired DNA fragments were synthesized and inserted into pEX-A128 and pEX-A2, respectively (MWG-Biotech, Ebersberg, Germany). The first DNA fragment comprised the 232-bp *pilA* promoter carrying the *A. olearius nifH* Shine-Dalgarno sequence and its spacer region (110), followed by the unmodified ATG-start codon, I end, and 118 bp of the 5′ end of *tnp*. The second DNA fragment covered 356 bp of the 5′ end of *npt* and the adjacent transposon sequence, which was originally flanked by *npt* and the *xylX* promoter. The two fragments were joined by inserting the first one into the pEX-A2 variant via NdeI/HindIII restriction sites. Successful cloning was proven by restriction analysis and PCR. To yield the *A. olearius*-specific transposon delivery plasmid pXMCS2::Tn*5PpilA*, the joined fragments were cloned into the promoterless pXMCS2::Tn*5Pxyl* backbone using KspAI/EcoNI restriction sites. The constructed plasmid was verified by restriction analysis and Sanger sequencing (LGC Genomics, Berlin, Germany). To guarantee DNA diversity for cluster identification and accurate base calling during Illumina Sequencing, transposon O-end modifications were carried out. Via the O-end-located SapI restriction site, a 20-bp DNA linker sequence providing NcoI/BbsI restriction sites was inserted. Generation of 20 pXMCS2::Tn*5PpilA* variants differing in a 14-bp barcode sequence was achieved by inserting 37-bp DNA fragments into the NcoI/BbsI restriction sites. Correctness of DNA sequences was proven by restriction analysis and Sanger sequencing (LGC Genomics, Berlin, Germany).

### Generation and storage of transposon mutants.

For the generation of the Tn*5* mutant master library, five independent electroporation experiments were performed each yielding approximately 120,000 Tn*5* mutant colonies. The pXMCS2*::Tn5PpilA* variants were independently isolated from the plasmid-harboring E. coli strains by using the Monarch plasmid miniprep kit (New England Biolabs, Frankfurt am Main, Germany) according to the manufacturer’s instructions. Plasmid DNA was eluted in 30 μL of H_2_O, pooled to equal concentrations dialyzed against water, and used for electroporation. *A. olearius* wild-type cells for electroporation were precultured in liquid VM-malate medium at 30°C to an OD_578_ of 1.0 to 1.5. The main cultures were inoculated to an OD_578_ of 0.05 and grown in VM-malate at 37°C until an OD_578_ of 0.5 was reached. Cells were cooled for 30 min at 4°C, followed by two cycles of centrifugation and washing, to be finally stored in 10% glycerol prior to electroporation. Transposition events were selected on VM-malate agar plates supplemented with kanamycin at 90 μg/mL. Growth of transposon mutants was restricted to 3 days at 37°C. Approximately 2,500 CFU were pooled and resuspended in VM-malate medium, and cell suspensions were adjusted to an OD_578_ of 30.

Mutant pools were stored individually in 10% dimethyl sulfoxide (DMSO) at a final OD_578_ of 15 in a 96-well plate at −80°C. Additionally, all pools were combined and the respective sublibrary stored in 10% DMSO at a final OD_578_ of 1.5 in 2-mL cryotubes at −80°C. The final *A. olearius* Tn*5* master library consisted of five Tn*5* mutant sublibraries comprising in total ~6 × 10^5^ transposon mutants.

### Library passage and selective growth conditions.

For selective growth, first the mutants were revived after storage. Two 2-mL aliquots of each Tn*5* mutant sublibrary were collected in 30 mL VM-malate medium and thawed. Cells were harvested by centrifugation at 3,220 × *g* for 10 min and resuspended in VM-malate. Diluted to an OD_578_ of 0.1, Tn*5* mutants were grown at 37°C in VM-malate until an OD_578_ of 0.3 was reached. For genomic definition of the current Tn*5* mutant pool, aliquots of the revived cells were taken, and cell pellets were stored at −20°C for genomic DNA (gDNA) isolation. Prior to bioreactor inoculation, revived cells were pelleted by centrifugation at 3,220 × *g* for 10 min and resuspended in SM. Following two cycles of centrifugation and resuspension, cells were finally diluted in SM. Growth in the bioreactor was carried out in a 1.5-L volume starting with an OD_578_ of 0.0078. Tn*5* mutant cells were collected by centrifugation at an OD_578_ of 1.0 and subsequently stored at −20°C for gDNA isolation.

### DNA preparation and amplification.

Phenol-chloroform extraction for DNA purification was implemented with minor changes to the standard protocol (111). For cell lysis and protein denaturation, sodium dodecyl sulfate (SDS [5% in H_2_O]) was applied instead of *N*-lauroylsarcosine solution. Four cycles of Tris-EDTA (TE)-buffered phenol extraction were carried out embedding RNA degradation by adding 5 μL RNase A (10 mg/mL) and incubating for 30 min at 37°C. The final DNA extraction was carried out with TE-buffered chloroform. After precipitation of DNA with isopropanol, pellets were diluted in 20 μL of TE buffer and stored at −80°C. Each sublibrary of the Tn*5* mutant master library was divided into four pools with DNA extractions for each pool carried out in triplicates. For this, cells stored in 96-well plates were thawed on ice and pools of ~30,000 Tn*5* mutants created. In the case of Tn*5* mutant cells representing master library composition prior and after selective growth, three aliquots of each mutant population were processed in parallel and DNA extracted in triplicates.

For determination of Tn*5* insertion sites by amplification between the Tn*5* O end and the adjacent genomic DNA, the protocol was based on a previously described nested-PCR protocol (32). DNA quality (260/280 ≤ 2.0) and quantity (≥100 ng/μL) were first evaluated spectrophotometrically with a NanoDrop 2000 (Thermo Scientific, St. Leon-Rot, Germany) and Quantus fluorometer (Promega, Madison, WI, USA), respectively. DNA at 100 ng/μL in 1 μL was added to each reaction mixture by employing DreamTaq DNA polymerase (New England Biolabs, Frankfurt am Main, Germany) in 50-μL reaction volumes (for primer information, see below). Per sample, three PCRs were carried out, combining the transposon-specific primer with one of the three semiarbitrary primers. The thermocycling parameters are listed below: 94°C for 3 min, followed by 7 cycles of 94°C for 0.5 min, 42°C for 0.5 min declining by 1°C per cycle, and 72°C for 1 min, followed by 26 cycles at 94°C for 0.5 min, 58°C for 0.5 min, and 72°C for 1 min, and ending with a final elongation at 72°C for 3 min. In the subsequent second PCR, Illumina-compatible adapter sequences as well as a sample-specific index sequences were introduced by employing the *Illumina P5* primer and the respective *Illumina P7 index* primer. Individual reactions were supplemented with 2 μL of the first PCR product and cycled under the following thermal conditions: 94°C for 3 min, followed by 31 cycles at 94°C for 0.5 min, 64°C for 0.5 min, and 72°C for 1 min, followed by a final step at 72°C for 3 min.

### Illumina sequencing.

PCR products were size selected and purified, aiming for an approximate fragment size of 450 to 750 bp. Amplicons were subjected to agarose gel electrophoresis and subsequent DNA purification with the Monarch DNA gel extraction kit (New England Biolabs, Frankfurt am Main, Germany). According to the manufacturer’s instructions, the Ampure XP system (Beckman Coulter, Brea, CA, USA) was applied in a 1:1.8 volume ratio for a stringent removal of contaminants, followed by a second size selection aiming for a mean fragment size of 550 bp and a final double cleanup in a 1:1 volume ratio. DNA quality was determined with a NanoDrop 2000 (Thermo Scientific, St. Leon-Rot, Germany), and DNA quantities were measured with a Quantus fluorometer (Promega, Madison, WI, USA). Library fragment sizes were controlled by running 1-μL samples on the Agilent Bioanalyzer (Agilent Technologies, Santa Clara, CA, USA). Furthermore, the presence and quantity of functional library fragments were verified by quantitative PCR (qPCR) according to Illumina standard protocols. Final library concentrations were adjusted to 4 nmol/L. DNA sequencing with 150-bp read length was performed on a NextSeq 500 Illumina platform according to the manufacturer’s instructions. Using the NextSeq 500/550 v.2.5 sequencing reagent kit, a cluster density of 200,000 /mm^3^ was intended, and overall nucleotide diversity was addressed by a 20% PhiX control spike-in.

### Processing of raw reads.

Illumina FASTQ files were demultiplexed according to the employed barcode sequences. Raw reads that passed quality trimming using Trim-Galore (https://github.com/FelixKrueger/TrimGalore) were subsequently filtered for presence of the Tn*5* transposon sequence. Received Tn*5* insertion-specific reads were processed and analyzed by a custom Python script and custom Perl scripts as follows. After conversion of FASTQ files into FASTA files, sequences derived from the Tn*5* transposon or from the semiarbitrary primers were removed. Adjusted reads representing *A. olearius* genomic DNA sequence were aligned with the reference genome (7) using NCBI blast (112). Aligned sequences were filtered for a perfect match of the first 15 bases reading out of the Tn*5* O end to the reference genome (7) using custom Perl scripts. In case of multiple genomic hits, the hit with highest coverage and overall identity value was kept. The insertion site of the Tn*5* was calculated according to the start and endpoint of the BLAST results using custom Python scripts. Data were further processed with a custom R Tn*5* analytical pipeline. The first base of every trimmed read originated from the genomic sequence adjacent to the Tn*5* O end. Considering the orientation of the *A. olearius pilA* promoter, the corresponding Tn*5* insertion site was by definition the first base adjacent to the Tn*5* I end. This site correction considered the Tn*5* characteristic 9-bp duplication and the change in genomic orientation. Read counts were generated. Finally, data sets were reduced listing unique insertion sites only.

### Analysis of putatively essential genes and depletion analysis.

Analysis of putatively essential genes was performed using a custom-written R script. First, Tn*5* insertion sites were assigned to the *A. olearius* ORF sequences based on the published KEGG (Kyoto Encyclopedia of Genes and Genomes) reference genome and determination of insertion orientation (sense or antisense). Sense insertions in the +1 reading frame of an ORF are expected to drive Tn*5PpilA*-directed target gene transcription. Activity of the *pilA* promoter gives rise to an engineered mRNA carrying the Tn*5*-derived ATG codon, SD, and I-end sequence, followed by the initial but 5′-depleted ORF sequence. While integrity of transcriptional units is preserved, synthesis of a functional albeit truncated protein or posttranslational reassembly of protein subunits cannot be ruled out. For this reason, insertion sites in the +1 reading frame of an ORF were neglected and not further analyzed. Additionally, insertions located in the stop codon and sense/antisense insertions in the last/first 9 bp of an ORF were not considered. Pursuing a conservative analysis, the following stringent criteria were applied. Genes with ORFs devoid of any insertion site were defined as non-hit genes and ergo putatively essential. Furthermore, genes displaying insertions only located in the last 10% of an ORF or an insertion-free gap of at least 80% of ORF length were assigned as putatively essential. The described thresholds were evaluated in a sensitivity analysis by determining the number of genes passing filtering and set regarding the aim of lowering the risk for potential false positives.

For analysis of consecutive putatively essential protein-coding genes, first the probability of *n* putatively essential protein-coding genes clustering in the *A. olearius* genome just by chance was determined by a permutation test. Therefore, the complete observed sequence of putatively essential and nonessential protein-coding genes was permutated 100,000 times, and *P* values for sequences of consecutive putatively essential protein-coding genes of length *n* were calculated. Based on the Bonferroni-Holm method for controlling the family-wise error rate with a significance level of α = 0.05, consecutive putatively essential protein-coding genes of length *n* ≥ 6 were found to be statistically significant. Genomic sections and corresponding insertion sites were visualized employing a custom-written R script.

For enrichment and depletion analysis, raw sequence reads were first processed as described above. Insertions located in the +1 reading frame and in the stop codon and sense/antisense insertions in the last/first 9 bp of an ORF were filtered out. Input data sets were generated as count matrices, giving the sum of read counts per gene in a sample. For pairwise comparisons, genes previously described as putatively essential were neglected and excluded from further analysis. Normalization of read counts over samples was done applying the DESeq2 package-embedded “median of ratios” method (60). Read counts were modeled by DESeq2, fitting a negative binomial generalized linear model. Via the package-embedded Wald test, gene specific *P* values were determined. For multiple testing, the DESeq2-embedded Benjamini-Hochberg method with a significance level of α = 0.1 was applied.

### Data availability.

Scripts used in the analysis are provided in a data repository: https://github.com/TheresaHarten/Azoarcus_TnSeq. The raw data have been deposited in the SRA archive (https://www.ncbi.nlm.nih.gov/sra) under accession no. PRJNA889402.
