# Supplementary material for: Elucidation of Essential Genes and Mutant Fitness during Adaptation toward Nitrogen Fixation Conditions in the Endophyte Azoarcus olearius BH72 Revealed by Tn-Seq
Source: Microbiol Spectr. 2022 Nov 23;10(6):e02162-22. doi: 10.1128/spectrum.02162-22 (PMC9769520; doi:10.1128/spectrum.02162-22)
Supplement: Supplemental file 4 — Fig. S1-S6. Download spectrum.02162-22-s0004.pdf, PDF file, 1.6 MB [file spectrum.02162-22-s0004.pdf]

Supplementary Figures Harten *et al.*

Elucidation of Essential Genes and Mutant Fitness during Adaptation towards Nitrogen Fixation Conditions in the Endophyte *Azoarcus olearius* BH72 Revealed by Tn-Seq

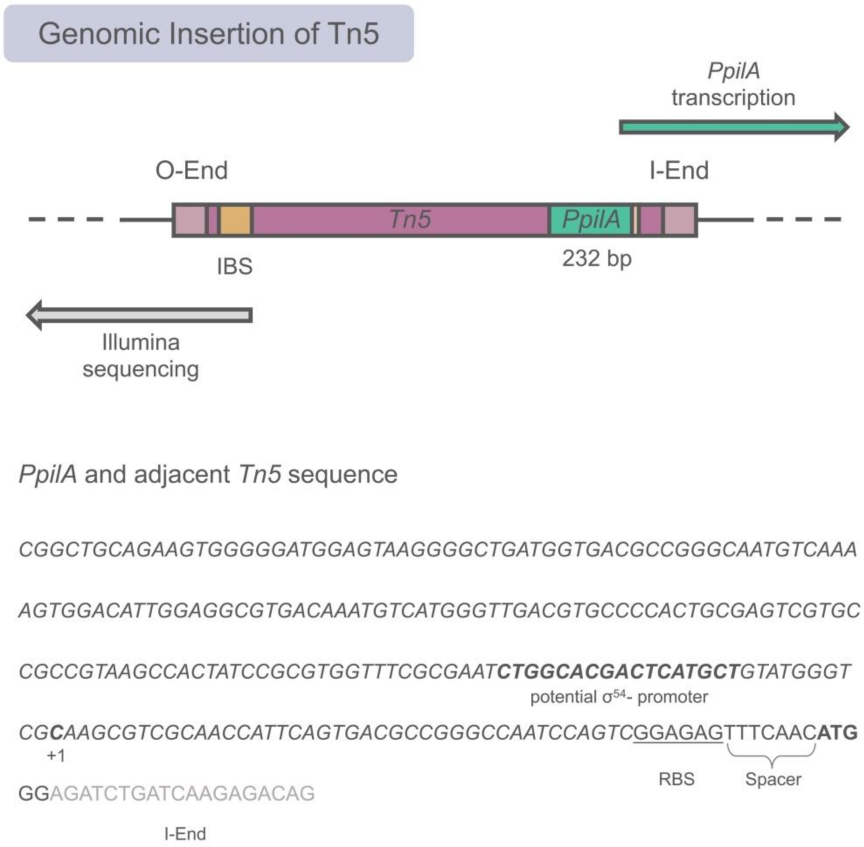

FIG S1. The *Tn5PpilA* transposon: Schematic drawing of the inserted Tn5 transposon flanked by the characteristic O-End and I-End region. While the O-End bears the Illumina-primer binding site (IBS), the I-End carries the *A. olearius* native *pilA* promoter sequence (*PpilA*). The *pilA* promoter, spacer and I-End sequence are shown: *pilA* sequence with its predicted σ^{54} and ribosomal binding site (RBS), the introduced spacer region, start codon (ATG) and I-End in light grey.

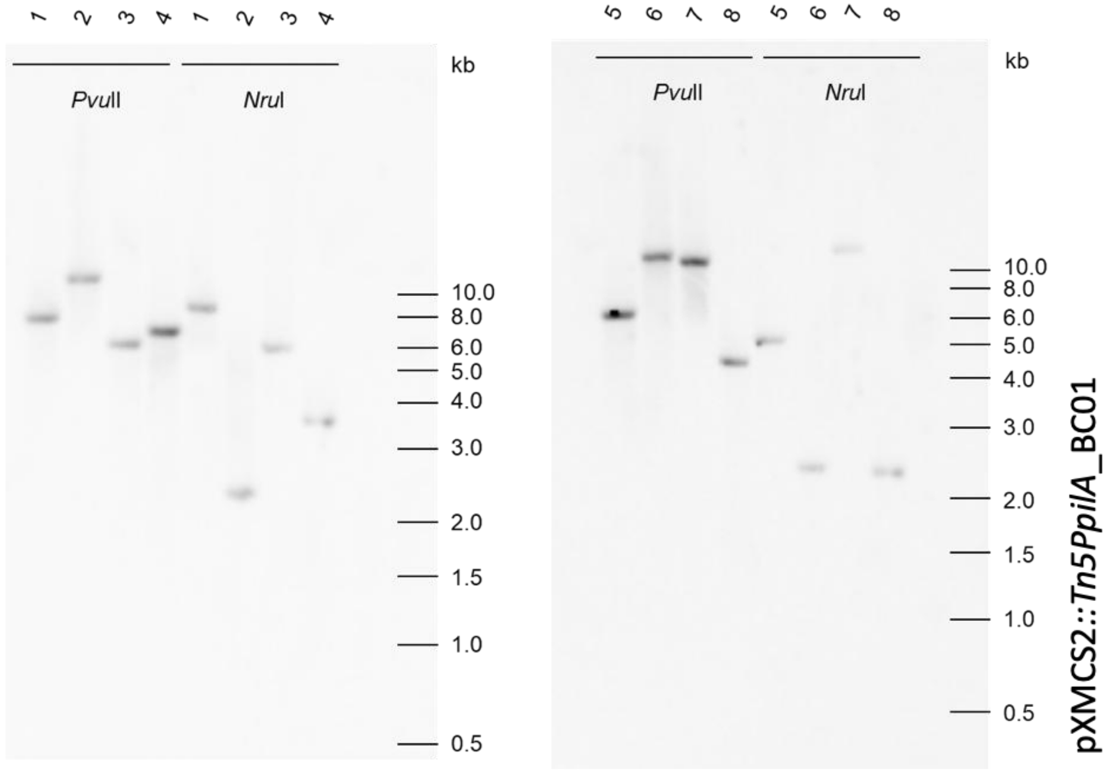

FIG S2 Analysis of randomly selected Tn5 mutants by Southern hybridization of genomic DNA verified *Tn5PpilA* functionality; insertions occurred randomly and singularly in the *A. olearius* genome. Two different restriction endonucleases used on mutants 1 – 8. DNA size marker given in kb.

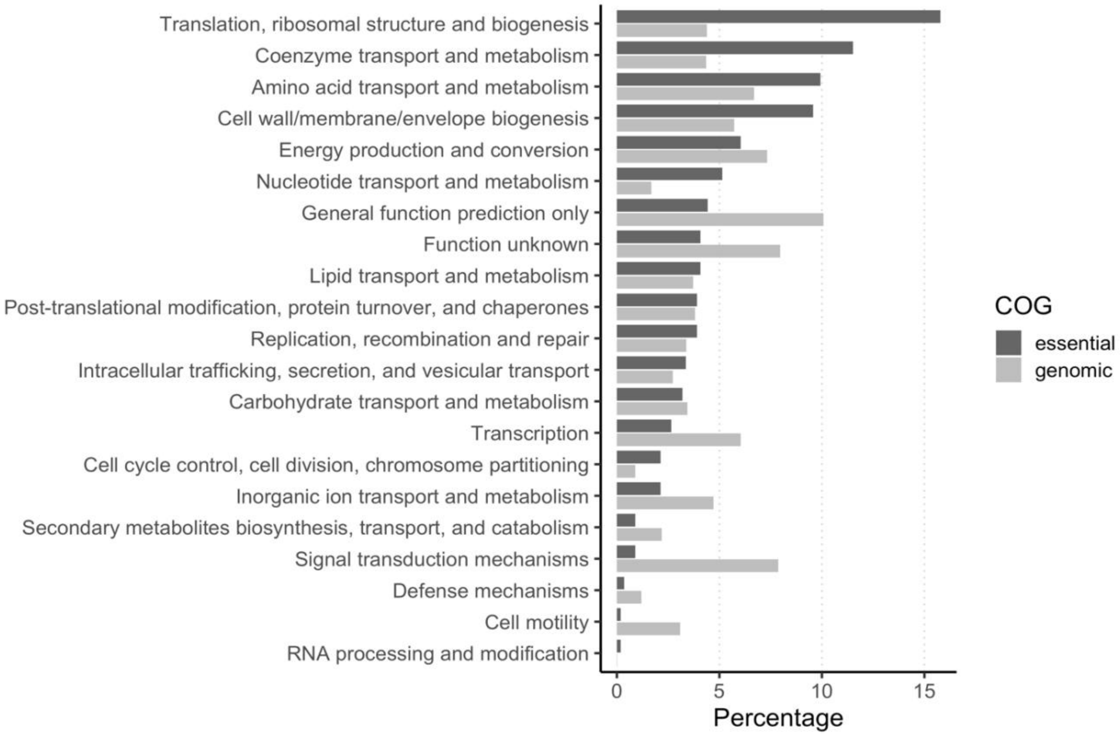

FIG S3 Comparison of the representation of assigned COGs of the putative essential and genomic gene set. Bars indicate representation of each COG among the essential and genomic gene set, respectively. Genes with more than one assigned COG were accordingly counted.

The group "coenzyme transport and metabolism (H)" was found to be highly enriched. Among the encoded pathways riboflavin (B_2) biosynthesis and subsequent production of flavin mononucleotide (FMN) and flavin adenine dinucleotide (FAD) were defined as putative essential (*ribH*, *ribE*, *ribF*, *ribD*). Furthermore, we report the cobalamin (B_{12}) biosynthetic genes *azo2781*, *azo3517*, *cobD2*, *cobA2B*, *cobU*, *cobTS* and the related cluster of *azo3529*, *cbiXCDEL*, *cbiGaGbH* as well as related *gltX* and *hem* genes to be putative essential, concordantly also all further steps to protoheme. In the groups "amino acid transport and metabolism" (E) and "nucleotide transport and metabolism" (F), genes related to arginine and histidine biosynthesis and to the linked pyrimidine and purine metabolisms were observed. In "cell wall/membrane/envelope biogenesis" (M), genes related to peptidoglucan biosynthesis (*mur*) and lipopolysaccharide metabolism (*waa*, *lpx*) were essential. Lastly, TonB system (*exbD3B2*, *exbD4*) and protein export (*sec*, *tat*) related genes belonging to the group "intracellular trafficking, secretion, and vesicular transport" (U) were detected.

A

B

C

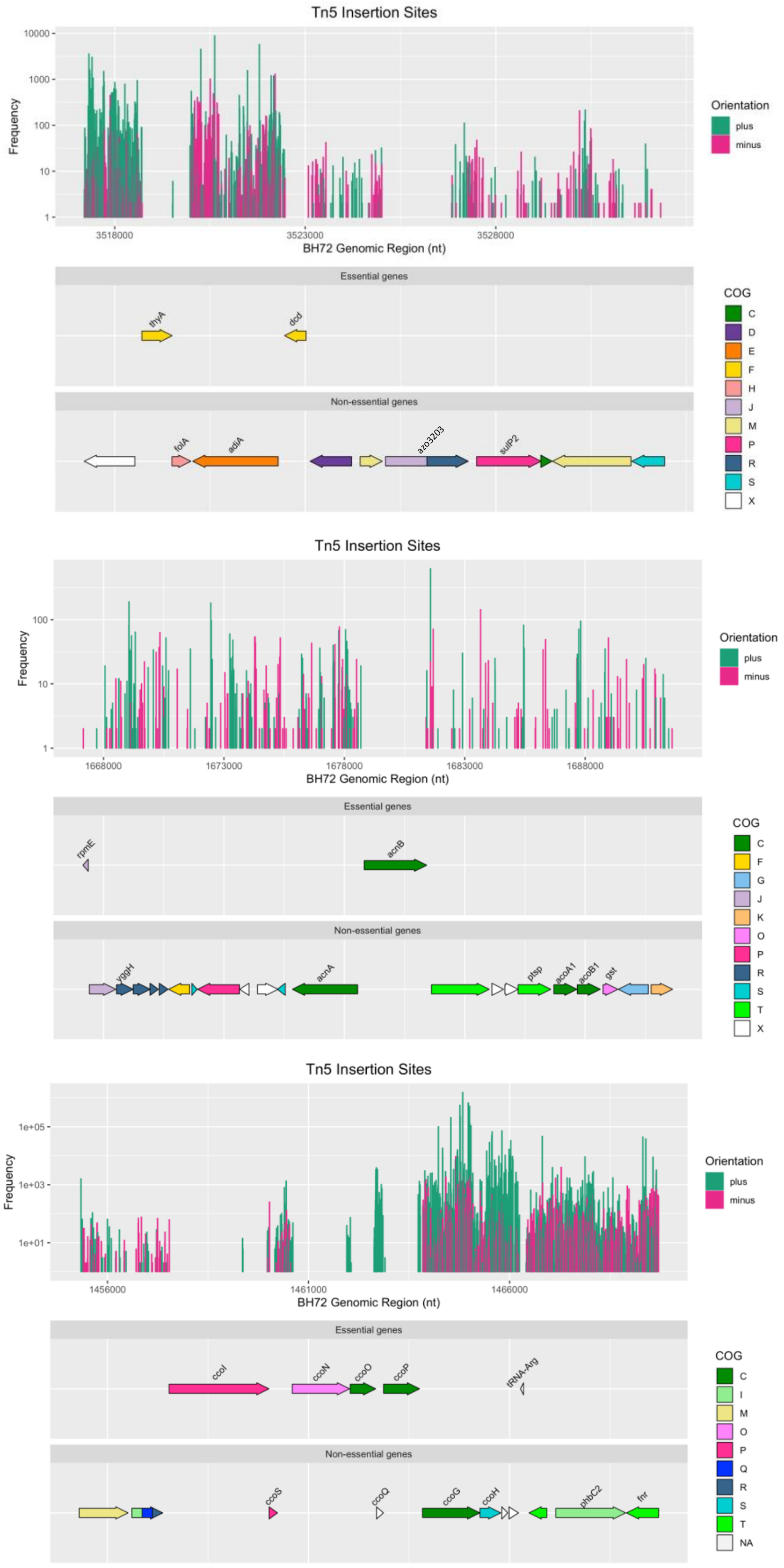

Tn5 Insertion Sites
Frequency
BH72 Genomic Region (nt)
Orientation
plus
minus
Essential genes
Non-essential genes
COG
3518000
3523000
3528000
thyA
dcd
folA
adiA
azo3203
sulP2
1668000
1673000
1678000
1683000
1688000
rpmE
acnB
yggH
acnA
ptsp
acoA1
acoB1
gst
1456000
1461000
1466000
ccoI
ccoN
ccoO
ccoP
tRNA-Arg
ccoS
ccoQ
ccoG
ccoH
phbC2
fnr

FIG S4 Insertion sites in genomic region of *azo3203* (A), *acnA* and *acnB* (B) and the *ccoNOQP* cluster (C) of *A. olearius*. Upper to lower panel: frequency of detected Tn5 insertion sites of the respective genomic region and the determined insertion orientation, green; plus, pink; minus, genes defined as putative essential and as putative non-essential and their assigned COG (NA, no COG assigned). (A) *azo3203* encoding the methionyl-tRNA synthetase. (B) *acnA* and *acnB*, both genes encode probable aconitate hydratase enzymes involved in the TCA cycle. While *acnB* lacks insertions, *acnA* shows a high insertion density. (C) *ccoNOQP* cluster encoding a high affinity cbb_3-type cytochrome *c* oxidase.

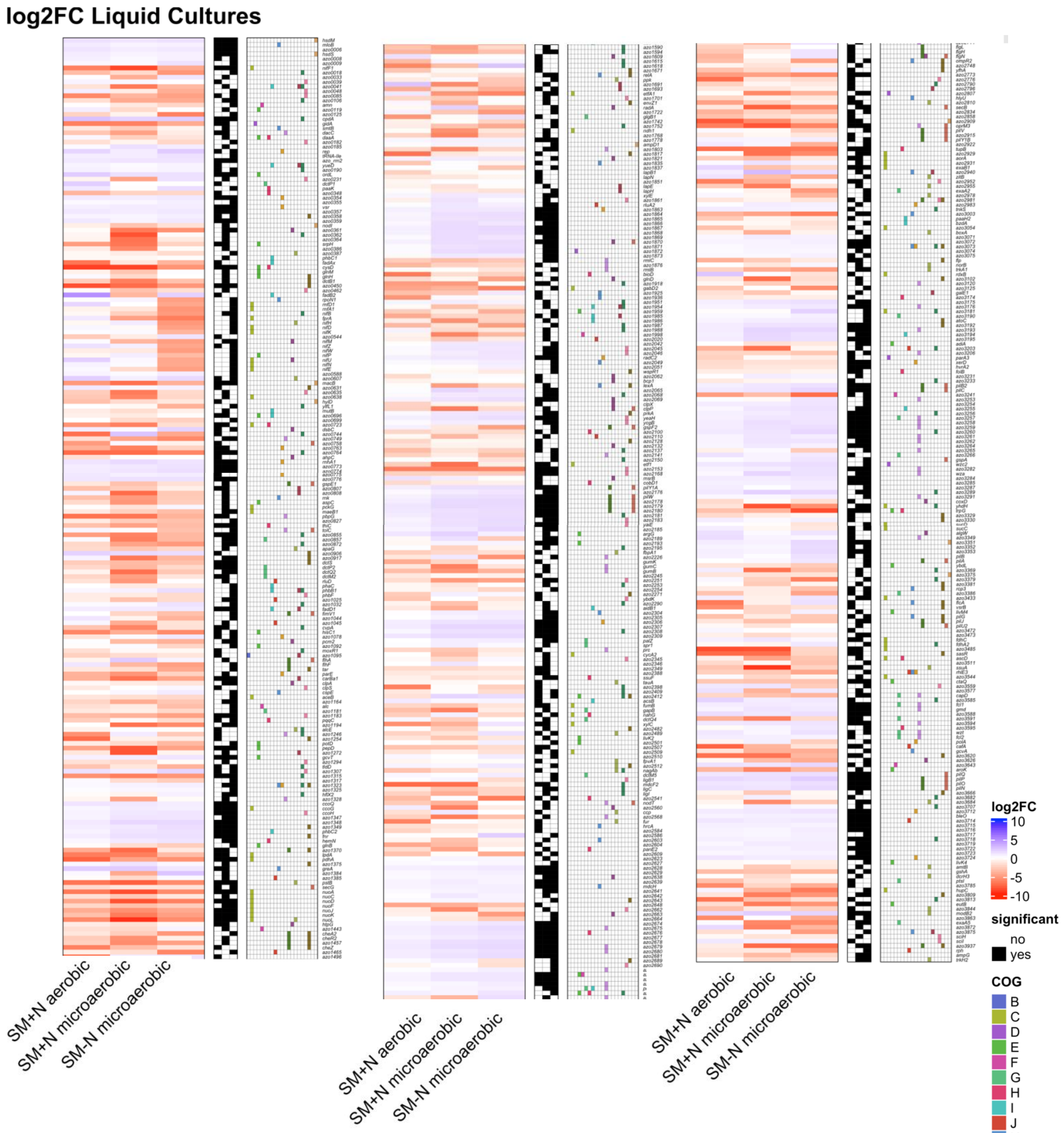

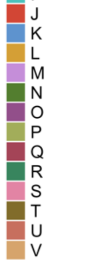

FIG S5 Comparison of genes identified as significantly depleted or enriched upon mutation during selective growth. Left to right panel: heat map of genes identified as significantly depleted or enriched upon mutation for at least one of the tested conditions, in the order as they appear in the genome. The level of change in abundance compared to the master library is given by negative (depleted) and positive (enriched) log2FC; overview of significance, black; significant, white; not significant; COG information of presented genes.

A

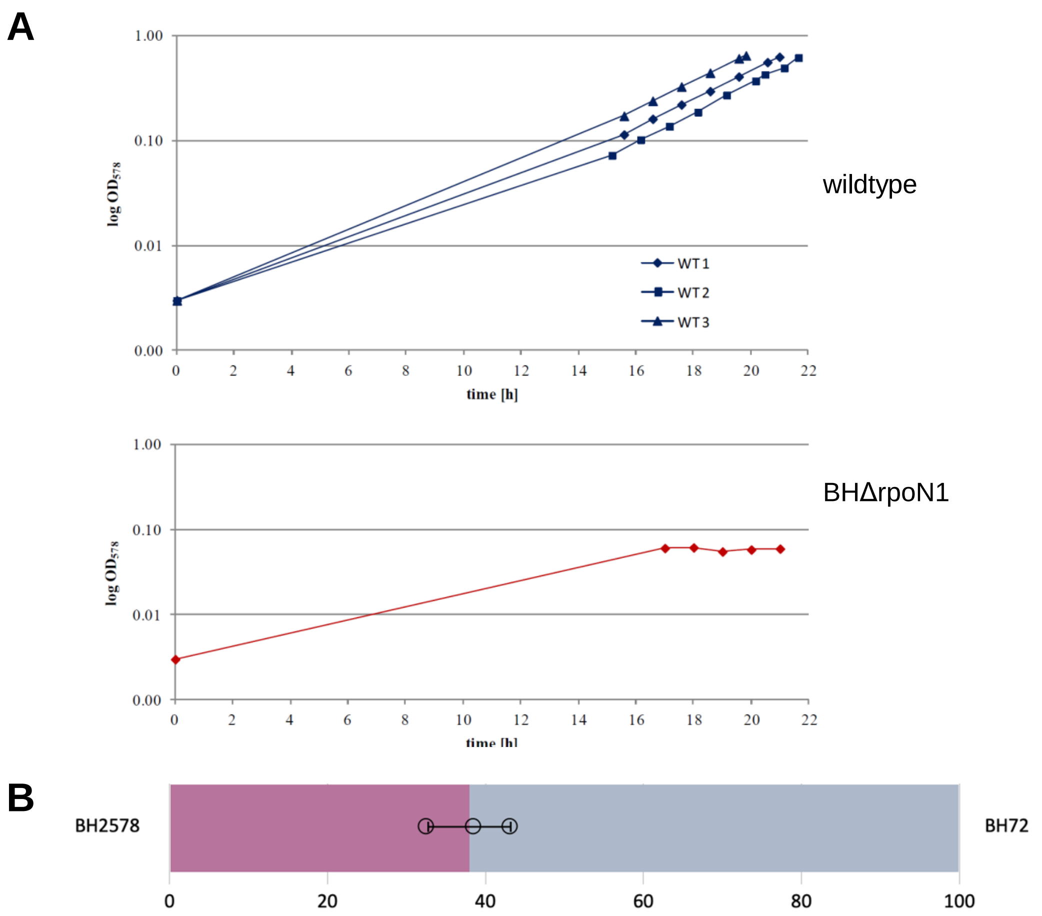

FIG S6 Effect of mutations in candidate genes on fitness of *A olearius.* (A) Growth of *A. olearius* BH72 wildtype or mutant BHΔrpoN1, respectively, under conditions of nitrogen fixation in bioreactor. Strain BH72 was grown in nitrogen-free SM-N medium under microaerobic conditions (O_2 0.3 %) for 20 h to 22 h in an oxygen-controlled bioreactor. The starting OD_{578} was 0.003 and the first OD measurement was performed after around 15 h. In frame-deletion mutant BHΔrpoN1 was grown under the same conditions, but with supplementation of glutamate (20 mM) to the medium. (B) Competitive growth of wild type BH72 and kanamycin-resistant gene inactivation mutant BH2578 under aerobic conditions on synthetic SM medium containing ammonium chloride. Strains were mixed at equal cell numbers, and were differentiated according to their antibiotic resistance before and after roughly 20 generations of exponential growth. Relative abundance in mixture after incubation is shown for three independent experiments.
